# A dataset revolutionizing Indian bay leaf analysis

**DOI:** 10.1016/j.dib.2024.111024

**Published:** 2024-10-16

**Authors:** Priyanka Paygude, Sandip Thite, Ajay Kumar, Amol Bhosle, Rajendra Pawar, Renuka Mane, Rahul Joshi, Manisha Kasar, Prashant Chavan, Milind Gayakwad

**Affiliations:** a Bharati Vidyapeeth (Deemed to be University) College of Engineering, Pune, India; b Vishwakarma University, Pune, India; c Department of Computer Science and Engineering, Manipal University, Jaipur, India; d MIT School of Computing, MIT Art Design and Technology University, Pune, India; e School of Computer Engineering and Technology, Dr Vishwanath Karad, MIT World Peace University, Pune, India; f Symbiosis Institute of Technology, Symbiosis International (Deemed University), Pune, India

**Keywords:** Classification, Indian bay leaf dataset, Machine learning, Indian bay leaf quality assessment

## Abstract

Indian bay leaf is a crucial spice in Indian cuisine. However, its quality and authenticity are often compromised. To address this, we introduce The Digital Indian Bay leaf dataset, a comprehensive collection of high-resolution 5696 images capturing diverse bay leaf samples under controlled conditions. The dataset encompasses variations in leaf conditions such as fresh leaf, dried leaf and diseased prone leaf. The dataset is meticulously curated to support research in condition analysis and machine learning applications for leaf quality assessment. To ensure data diversity, each category includes a wide range of images captured under controlled conditions with varying lighting, background, and leaf orientation. By providing a standardized and accessible resource, this dataset aims to accelerate research in this domain and contribute to the improvement of the Indian spice industry.

Specifications Table

**Table:** 

Subject	Machine Learning, Food Science, Agronomy and Crop Science
Specific subject area	Indian Bay Leaves dataset for classification, identification and quality assessment
Type of data	Image
Data collection	This dataset contains high-resolution images of Indian bay leaves (Cinnamomum Tamala) categorized into three folders for classification tasks. Healthy Leaf folder contains images of fresh, green Indian bay leaves without any visible damage or disease. Dried Leaf folder contains images of dried Indian bay leaves, commonly used in cooking. The leaves may appear brown or slightly lighter depending on the drying process. Diseased Leaf folder contains images of Indian bay leaves exhibiting signs of disease. These may include discoloration, spots, wilting, or other abnormalities. We took meticulous care to capture the leaves under different lighting conditions, and with a variety of backgrounds, ensuring a rich and diverse collection. Each individual dry type of leaves was captured under controlled lighting conditions to ensure consistent image quality. Images were saved in JPEG format and resized to a uniform resolution of 1080 × 1920 pixels for efficient storage and processing. Images were renamed sequentially for clear organization within the dataset.
Data source location	*At Post Donje, Taluka Haveli, District Pune, Maharashtra, India* Longitude and Latitude: 18.3999° N, 73.7860° E
Data accessibility	Repository name: Indian Bay Leaves or Cinnamomum Tamala Leaves Dataset Data identification number: 10.17632/s9t7sr52wg.2 Direct URL to data: https://data.mendeley.com/datasets/s9t7sr52wg/2 Instructions for accessing these data:
Related research article	*None*

## 1 Value of the Data

• The Indian Bay Leaf, or Tejpat (as it's called in Hindi), comes from the Cinnamomum Tamala tree. This aromatic tree, native to India and several other Asian countries, can grow up to 20 m tall. Its leaves, with a distinctive clove-like aroma and a hint of pepper, are widely used in both cooking and traditional medicine. Interestingly, these leaves are believed to be one of the main sources of the mysterious “malabathrum” mentioned in ancient texts.
• Dried leaves of Indian Bay leaf are used as flavoring agent in cooking. These leaves have medicinal properties, and it is also used as an adjunct therapy in diabetes.
• This dataset can be used to train machine learning models to automatically identify fresh, dried, and spoiled Indian bay leaves. This allows for faster and more consistent quality control in the food industry. The dataset can help identify leaves from other plants that may be fraudulently mixed with Indian bay leaves.
• The dataset contributes to the development of more robust computer vision algorithms for object recognition and classification, benefiting applications beyond Indian Bay leaves.

## 2 Background

India is a leading producer and exporter of bay leaves. The report highlights the increasing demand for bay leaves in the food, medicine, and beverage industry, the rising popularity of ethnic cuisines, and the growing awareness of health benefits associated with bay leaves as key factors driving the market growth.

This dataset can fuel the creation of smartphone apps that use image recognition to help consumers identify the quality of bay leaves at the point of purchase. The dataset can aid in developing machine learning models to detect leaves from other plants that might be fraudulently mixed with Indian bay leaves. This safeguards consumers and ensures the authenticity of the product. By training models on the dataset, researchers can contribute to the development of more robust computer vision algorithms for object recognition and classification tasks [[1], [2]]*.*

## 3 Data Description

To help machines better identify the condition of bay leaves, researchers created a dataset of dried, fresh, and diseased leaves. This collection of high-resolution images (1080 × 1920 pixels at 72 dpi) comes from Donje Village, Pune, and captures the leaves in various backgrounds, lighting, and at different angles. This variety ensures the algorithms trained on this dataset will be more adaptable to real-world scenarios.

**Fig. 1:**
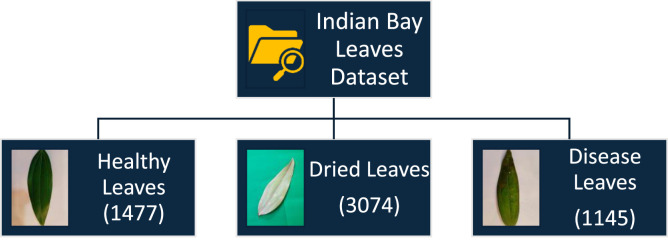
Organization of Dataset.

The entire dataset, which is organized into three folders, contains 5696 images of Indian Bay leaves. These images are stored in distinct folders for simple organization. 1145 photos of healthy fresh Indian bay leaves can be found in the first folder [[3], [4], [5]]. The second folder has a 3074 number of dried pictures of vibrant Indian bay leaves, which are frequently used as a spice in cooking. 1477 quantity of disease-prone Indian Bay leaves are kept in the third folder. The pictures were taken with a plain background, but under different lighting conditions, both indoors and outdoors. Fig. 1 shows the organization of the dataset [6].

## 4 Experimental Design, Materials and Methods

### 4.1 Experimental design

**Fig. 2:**
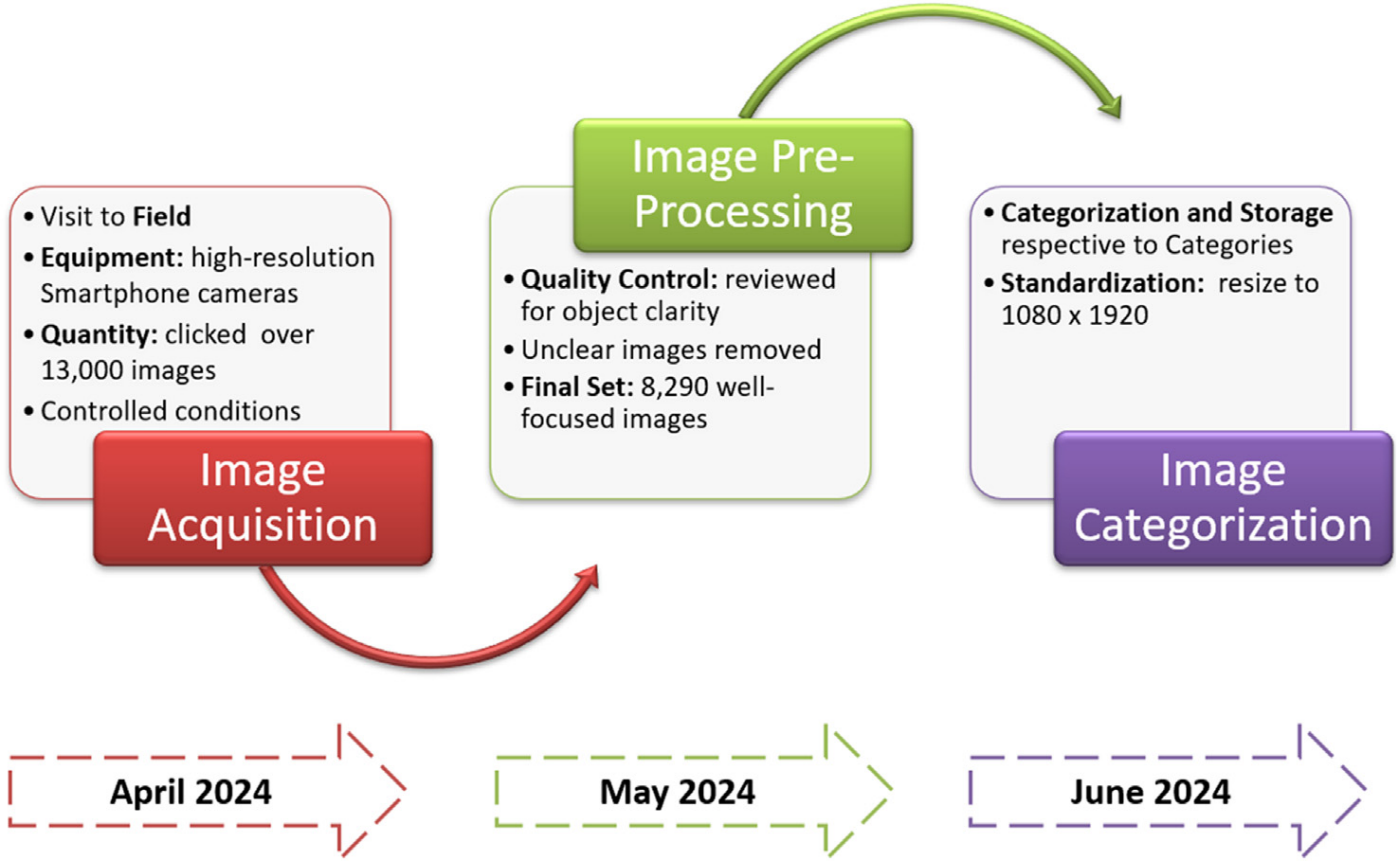
Data acquisition steps.

The Bay Leaf Disease dataset was created using high-resolution rear cameras of Realme 6i mobile phone, as detailed in Fig. 2. The process involved three key phases:

Phase 1: Image Acquisition (April):

• Field trips were conducted during the day to collect the leaves in a variety of conditions.
• Fresh healthy leaves are dried in natural sunlight to preserve their originality for a week.
• Capture images of bay leaves in its fresh, dried format. Also, images were captured for disease prone leaves.

Phase 2: Image Preprocessing (May):

• Collected photos were reviewed, and high-quality images were selected for the dataset.
• Chosen images underwent preprocessing steps like cropping, resizing, and enhancement as needed.

Phase 3: Image Categorization (June):

• This phase focused on organizing the collected images into distinct disease categories. It's crucial for building a usable dataset. The process involved two main steps:

3.1 Batch Conversion and Unique IDs: Images were converted to a JPEG format suitable for the dataset. Each image received a unique identifier for easy tracking and reference.

Each image was meticulously categorized according to the specific bay leaf disease it depicted. Table 1 shows sample images of each category.

**Table 1:** Sample Images of each category.

### 4.2 Materials

The camera apparatus used to take the images, and the parameters of the final images are described in this section:

Realme 6i: a. Make and Model: Mobile Realme 6i. b. Primary camera on the back: 48 MP, f/1.8

To ensure uniform image quality and compatibility throughout the dataset, every image that was recorded was converted to a standard resolution of 1080 × 1920 pixels and stored in JPEG format. The dataset is guaranteed to function flawlessly with a variety of machine learning applications thanks to this standardization process.

### 4.3 Methods

**Fig. 3:**
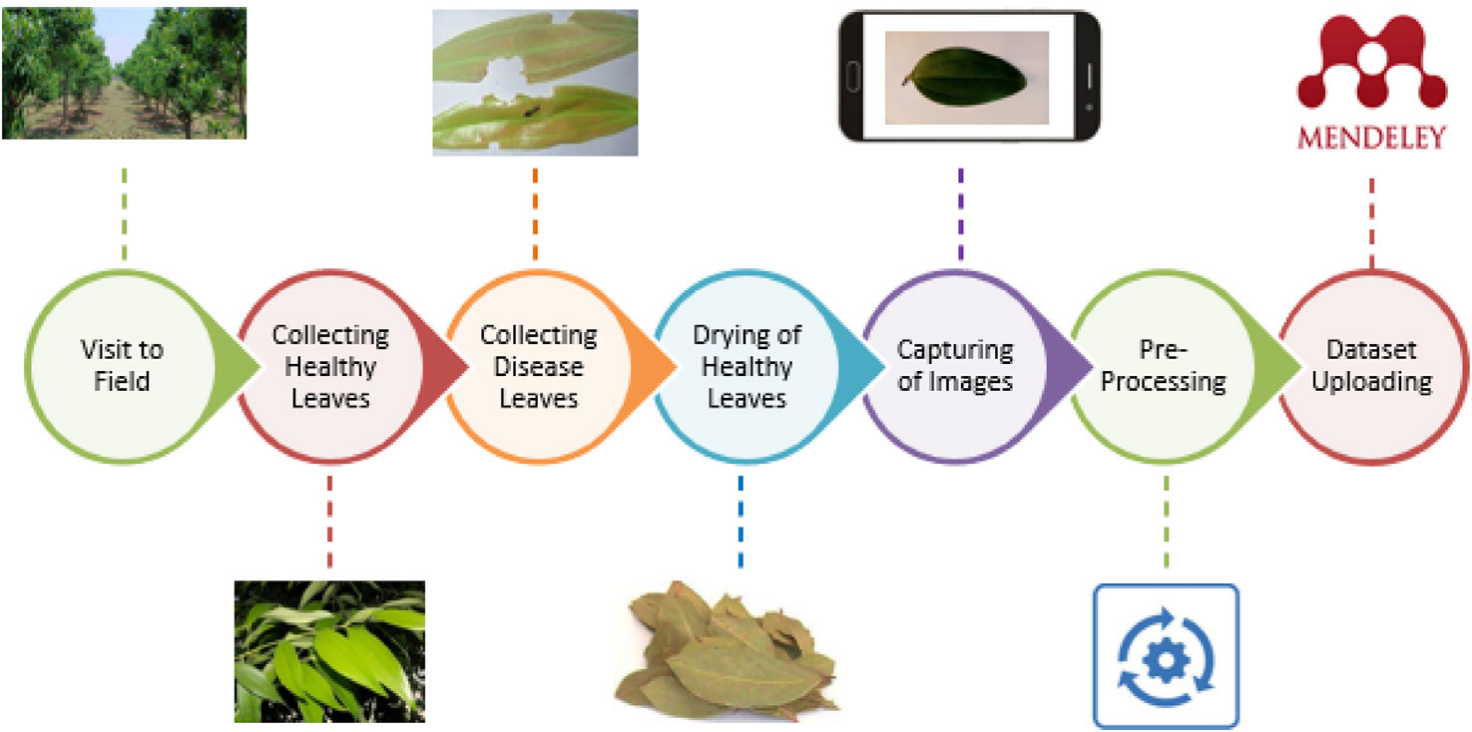
Stage by stage progress of dataset creation.

Field visits to farms that grow bay leaves, or tamala, were used to gather the dataset. The authors examined and took high-resolution photos of bay leaves in cooperation with farmers. Based on the existence of the disease and other characteristics, these photos underwent thorough classification, creating a methodically arranged dataset for study and investigation. Information was collected for the Bay Leaves Healthy and Disease dataset by visiting a farm located in Donje, Taluka-Haveli, District-Pune, India. Fig. 3 Stage by stage progress of Indian bay leaf dataset creation.

### 4.4 Highlighting the dataset's value

The provided Table 2 offers a comprehensive comparison of existing Indian bay leaf datasets across three distinct leaf conditions: healthy, dried, and diseased.

**Table 2:** Comparative table for Indian Bay Leaf Dataset.

Sr. No.	Dataset Ref. No.	Repository	No. of healthy Indian bay leaf Images	No. of Dried Indian Bay leaf Images	No. of Diseased Indian Bay leaf Images
1.	[7]	www.data.mendeley.com	Not Considered	1085	Not Considered
2.	[8]	https://universe.roboflow.com/	Not Considered	Not Considered	1120
3. (Our Dataset)	[9]	www.data.mendeley.com	1477	3074	1145

We tested the dataset's potential to improve the performance of pre-trained, well-known machine learning models in the classification of healthy and dried leaves using models like InceptionV3, Xception, and EfficientNetB0. Employing our dataset, these models were refined and their ability to accurately categorize different conditions of leaf was assessed. Pre-trained Model Performance on leaves condition analysis Task is evaluated in Table 3. The accuracy, Precision, Recall and F1 score attained by various pre-trained models after they were refined on the dataset picture dataset for the classification task are presented in this table, both pre- and post-training. Using this dataset as a strong training set, researchers can optimize these algorithms for higher accuracy.

**Table 3:** Comparison of ML models for pre-training and post-training over the dataset.

Machine Learning Model	Calculations before training on dataset				Calculations after training on dataset			
	Accuracy	Precision	Recall	F1 Score	Accuracy	Precision	Recall	F1 Score
**Xception**	38.18 %	42.66	58.18	0.48	91.23 %	95.31	95.31	0.95
**InceptionV3**	34.75 %	41.97	91.89	0.49	88.33 %	95.50	91.4	0.93
**EfficientNetB0**	38.57 %	50	46.87	0.48	93.53 %	94.23	98.99	0.96

## Limitations

The Indian Bay leaf or Cinnamomum Tamala Dataset mainly focus on fresh, dried and disease leaves in Pune, India region and lacks in further disease-based categorization of leaves.

## Ethics Statement

Our research aligns with Data in Brief's ethical considerations for datasets, as it does not involve animal or human subjects. Thus, confirm adherence to ethical considerations.

## CRediT authorship contribution statement

**Priyanka Paygude:** Conceptualization, Supervision, Writing – review & editing. **Sandip Thite:** Writing – review & editing. **Ajay Kumar:** Methodology. **Amol Bhosle:** Data curation, Writing – review & editing. **Rajendra Pawar:** Methodology. **Renuka Mane:** Writing – review & editing. **Rahul Joshi:** Writing – review & editing. **Manisha Kasar:** Data curation. **Prashant Chavan:** Writing – review & editing. **Milind Gayakwad:** Conceptualization.

## Data Availability

Mendeley DataIndian Bay Leaves dataset for classification and identification for quality assessment (Original data).
